# Regulation of Hindbrain Vascular Development by *rps20* in Zebrafish

**DOI:** 10.3390/cells14141070

**Published:** 2025-07-13

**Authors:** Xinyu Shen, Zhaozhi Wen, Shunze Deng, Yuxuan Qiu, Weijie Ma, Xinyue Dong, Jie Gong, Yu Zhang, Dong Liu, Bing Xu

**Affiliations:** School of Life Sciences, Nantong University, 9 Seyuan Road, Nantong 226019, China

**Keywords:** brain vasculature, *rps20*, vascular permeability, aging, zebrafish

## Abstract

During aging, the brain vasculature undergoes significant deterioration characterized by increased arterial tortuosity, compromised blood–brain barrier integrity, and reduced cerebral blood flow, all of which contribute to various neurological disorders. Thus, understanding the mechanisms underlying aging-related cerebrovascular defects is critical for developing strategies to alleviate aging-associated neurological diseases. In this study, we investigated the role of aging-related genes in brain vascular development using zebrafish as an in vivo model. By thoroughly analyzing scRNA-seq datasets of mid- and old-aged brain vascular endothelial cells (human/mouse), we found *ribosomal protein S20 (rps20)* significantly down-regulated during aging. qPCR analysis and whole-mount in situ hybridization validated a high expression of *rps20* during early zebrafish development, which progressively decreased in adult and aged zebrafish brains. Functional studies using the CRISPR/Cas9-mediated knockout of *rps20* revealed an impaired growth of central arteries in the hindbrain and a marked increased intracranial hemorrhage incidence. Mechanistically, qPCR analysis demonstrated a significant downregulation of *vegfa, cxcl12b, and cxcr4a,* key signaling molecules required for hindbrain vascular development, in *rps20*-deficient embryos. In conclusion, our findings demonstrate that *rps20* is essential for proper brain vascular development and the maintenance of vascular homeostasis in zebrafish, revealing a novel mechanism by which aging-related genes regulate brain vascular development. This study provides new insights that may aid in understanding and treating aging-associated vascular malformations and neurological pathologies.

## 1. Introduction

The vascular system is the first organ system to complete morphogenesis and achieve functional maturation during development, playing a pivotal role in sustaining embryogenesis and physiological activities [1,2]. The abnormal development or dysfunction of the vascular system is closely associated with cardiovascular diseases, which remain the leading cause of mortality worldwide [3]. The brain, as the most energy-consuming organ, is responsible for controlling sensory processing, behavior, and emotion [4,5]. To meet its high metabolic demands, the brain vascular system is highly ramified and efficient [6]. However, this cerebrovascular system becomes progressively compromised with aging, characterized by reduced vascular length and density, increased arterial tortuosity, impaired endothelial barrier function, heightened inflammatory responses, et al. [2,7]. These aging-induced cerebrovascular deteriorations are recognized as important contributors to the pathogenesis of neurodegenerative diseases, including vascular dementia, Alzheimer’s disease, Parkinson’s disease, et al. [2,8,9,10]. Therefore, elucidating the molecular mechanisms underlying aging-related vascular development and dysfunction holds significant promise for developing therapeutic strategies against these debilitating disorders.

Ribosomal proteins (RPs) are essential structural components of ribosomes, assembling with rRNAs to form functional ribosomes to facilitate the translation process [11]. *rps20* encodes the small ribosomal subunit protein uS10, a member of the ribosomal protein S10P family that constitutes the eukaryotic 40S subunit. Beyond its canonical ribosomal role, Rps20 also exhibits extra-ribosomal functions. For example, it can inhibit the activity of E3 ubiquitin ligase MDM2 through direct binding, thereby stabilizing the tumor suppressor P53 via post-transcriptional mechanisms [12]. Similar pleiotropic activities have been reported for other RPs, including Rpl5, Rpl11, Rpl23, etc. [13]. Emerging evidence has linked *rps20* to diverse biological processes, including pathogenesis, oncogenesis, cell proliferation, and viral protein synthesis [14,15,16]. However, its potential role in brain vascular development and the maintenance of cerebrovascular homeostasis remains unexplored.

In this study, we leveraged the optical transparency of zebrafish embryos to perform in vivo confocal imaging to investigate the roles of the aging-associated ribosomal protein *rps20* in brain vascular development, which will provide new insights into cerebrovascular diseases associated with aging.

## 2. Materials and Methods

### 2.1. Zebrafish Husbandry

Adult zebrafish were maintained at 28 °C with 14—10 h light–dark cycles. Zebrafish embryos were produced by pairwise mating, raised at 28.5 °C in 10-cm Petri dishes filled with egg water (6 g Instant Ocean/20 L RO water). Embryos used for live imaging after 24 hpf were treated with 0.004% phenylthiourea (PTU) in egg water to reduce pigmentation. Embryos and larvae were anesthetized using 3-aminobenzoic acid ester (Tricaine). Euthanasia was done with an overdose of Tricaine. The wild-type AB line and transgenic lines *Tg(fli1:GFP-caax)*, *Tg(flk1:eGFP)*, *Tg(fli1:GFP-caax; flk1:mApple-caax)* and *Tg(gata1:DsRed)* were used in this research. All animal studies were approved by Nantong University.

### 2.2. Bioinformatics Analysis

To identify potential genes involved in cerebrovascular aging, we reanalyzed previously published single-cell RNA sequencing data of brain endothelial cells from humans (5 mid- and 8 old-aged samples) and mice (3 mid- and 2 old-aged samples) [17,18,19]. This research employed an R language (v4.2.1)-based bioinformatics workflow within RStudio (v2024.09.0) integrating multiple open-source tools for data processing, statistical analysis, and visualization. Differentially Expressed Genes (DEGs) were identified using the FindMarkers function from the Seurat package, with significance thresholds set as follows: Human dataset: |log2FoldChange| > 1, adjusted *p*-value < 0.001, and expression in ≥20% of cells. Mouse dataset: log2FoldChange > 0 (up-regulated) or <0 (down-regulated), adjusted *p*-value < 0.001, and expression in ≥20% of cells. Gene Ontology (GO) and Kyoto Encyclopedia of Genes and Genomes (KEGG) enrichment analyses of DEGs were conducted using the clusterProfiler R package. The visualization of results, including volcano plots, violin plots, and chord diagrams, was performed using the EnhancedVolcano, ggplot2, and circlize packages. The R packages and versions are listed in Table 1.

### 2.3. Evolutionary Conservation Analysis of RPS20

The protein sequences of RPS20 from *Homo sapiens* (human), *Mus musculus* (mouse), *Rattus norvegicus* (rat), *Danio rerio* (zebrafish), *Xenopus tropicalis* (African clawed frog), and *Gallus gallus* (red junglefowl) were retrieved from UniProt. Multiple sequence alignment and homology analysis were performed using DNAMAN software. A phylogenetic tree was constructed in MEGA11 via the Neighbor-Joining method with 1000 bootstrap replicates. Additionally, spatial structures of human and zebrafish RPS20 (Rps20) proteins were predicted using AlphaFold under default parameters.

### 2.4. Whole-Mount In Situ Hybridization

The full-length coding sequence (CDS) of zebrafish *rps20* was amplified using primers Forward (5′-ATGGCATTTAAAGACACTGGCAAGG-3′) and Reverse (5′-TTAAGCATCTGCAATTGTGACC-3′). The purified CDS was ligated into the pGEM-T Easy Vector (Promega). Following single-restriction enzyme digestion, in vitro transcription and purification were performed to generate single-stranded nucleic acid probes targeting mature *rps20* mRNA. Zebrafish embryos at 1, 3, 5, and 7 days post-fertilization (dpf) were euthanized on ice (15–20 embryos per time point) and fixed overnight at 4 °C in 4% paraformaldehyde (PFA). After removing PFA, embryos were sequentially dehydrated in 25%, 50%, 75% methanol/PBST, and 100% methanol with 5-min incubations at each concentration and stored at −20 °C until use. After sequential processing steps—including rehydration, permeabilization, probe hybridization, and the removal of non-specifically bound probes via stringent washing—embryos underwent immunodetection with chromogenic visualization before final dehydration and optical clearing in a graded glycerol series. Images were acquired using an OLYMPUS MVX10 fluorescence stereomicroscope.

### 2.5. Quantitative Real-Time PCR

Total RNA was isolated from zebrafish tissues using Fastpure Total RNA Isolation Kit (Vazyme). Reverse transcription was performed with the HiScript IV All-in-One Ultra RT SuperMix for qPCR (Vazyme). Quantitative PCR (qPCR) reactions were prepared using the ChamQ Blue Universal SYBR qPCR Master Mix (Vazyme) and amplified on a 7500 Real-Time PCR System (Applied Biosystems). β-actin was selected as the reference gene. All qPCR primers used in this study are listed in Table 2.

For analyzing the gene expression of *vegfa*, *cxcr4a*, *cxcl12b*, *pcna*, *p15*, and *p53*, six batches of zebrafish samples were utilized. For each batch, 30 zebrafish embryos were collected at each of two developmental time points: 3 dpf and 5 dp. The expression profile of *rps20* in wild-type zebrafish brains across key developmental stages was characterized. Due to the technical challenges of dissecting brain tissue in early stages, whole-embryo samples were used for 1–7 days post-fertilization (dpf), while brains were isolated for later stages. For embryonic stages, samples were collected at 1, 3, 5, and 7 dpf, with 20 embryos collected per time point. For post-embryonic stages, 10 brains were collected at 1 and 5 mpf (month post-fertilization) per time point, 8 brains at 12 mpf, and 3 brains at 24 mpf. *rps20* expression levels in the zebrafish brains were determined in all collected samples.

### 2.6. O-Dianisidine Stain

This is a histochemical method that detects hemoglobin by leveraging its ability to oxidize *O*-dianisidine in the presence of hydrogen peroxide, resulting in a colored precipitate that marks erythrocytes [20]. Freshly prepared *O*-dianisidine staining solution (Table 3) was wrapped in aluminum foil to protect it from light and sonicated for 10 min until all components were fully dissolved. During the experiment, zebrafish were transferred into light-protected EP tubes, incubated with *O*-dianisidine staining solution at room temperature for 5 min, and then the staining solution was aspirated. Following this, 100% glycerol was added to the tubes. Zebrafish in glycerol were transferred to a glass dish and imaged under Nikon SMZ445 for observation and image acquisition.

### 2.7. Microinjection

The injection solution containing *rps20* sgRNA (CAAGGCUCCCGUUGAAGCCG) at 100 ng/μL and Cas9 protein (novoprotein-E365) at 500 ng/μL was loaded into borosilicate glass capillaries (World Precision Instruments, Sarasota, FL, USA) using a Micro Loader (Eppendorf, Hamburg, Germany). The capillary tip was manually adjusted to an optimal cusp with fine forceps. For microinjection, embryos were aligned in the agarose chamber, and the capillary tip was inserted into the animal pole of 1-cell stage embryos. A total volume of 1 nl of the solution was delivered per embryo. Post-injection, embryos were transferred to dishes and maintained at 28 °C in embryo medium, which was replaced daily. To preserve embryonic transparency, 0.003% PTU was added to the culture medium.

### 2.8. Confocal Imaging

Zebrafish larvae were anesthetized with Tricaine (SolarBio, Beijing, China). A 1% low-melting-point agarose solution (Invitrogen, Carlsbad, CA, USA) was prepared using culture medium and maintained at 40 °C. Zebrafish larvae were transferred to a glass-bottom culture dish (Cellvis, Sunnyvale, CA, USA), overlaid with the pre-warmed agarose solution, and carefully oriented to the desired position. Following agarose solidification, culture medium was added to the dish. The embedded zebrafish larvae were subsequently imaged using a Nikon A1R confocal microscope. For the brain vasculature development, the whole brain vasculature was imaged under a 20X objective lens (Nikon CFI Apochromat LWD Lambda S 20× WI, NA 0.95) or a 40X objective lens (Nikon CFI Apochromat LWD Lambda S 40× WI, NA 1.15). For the hind brain sprouting angiogenesis, time-lapse imaging was taken under a 20X objective lens with a 12-min interval.

### 2.9. Measurement of Body Length

Zebrafish larvae were immobilized and imbedded in glass-bottom dishes using 1% low-melting-point agarose gel and subsequently imaged with an MVX10 stereomicroscope (Olympus, Tokyo, Japan). The body length was measured by FIJI.

### 2.10. Quantification of Phenotype

The confocal images of the hindbrain vasculature were analyzed with FIJI to quantify the counts of CtAs derived from PHBC, counts of junctions between CtAs, and counts of junctions between CtAs and BA/PCS.

### 2.11. Statistical Analysis

All statistical analyses were performed using GraphPad Prism (version 9.5). One-way ANOVA with Tukey’s Honestly Significant Difference test was applied for multi-group comparisons, while unpaired Student’s *t*-tests were used for pairwise group analyses. Gene expression levels were evaluated through multiple unpaired *t-*tests. Statistical significance was denoted as follows: * *p* < 0.05, ** *p* < 0.01, *** *p* < 0.001, with ns (no significance) indicating *p* > 0.05.

## 3. Results

### 3.1. RPS20 Is Conservatively Down-Regulated in Aged Brain Endothelial Cells

**Figure 1:**
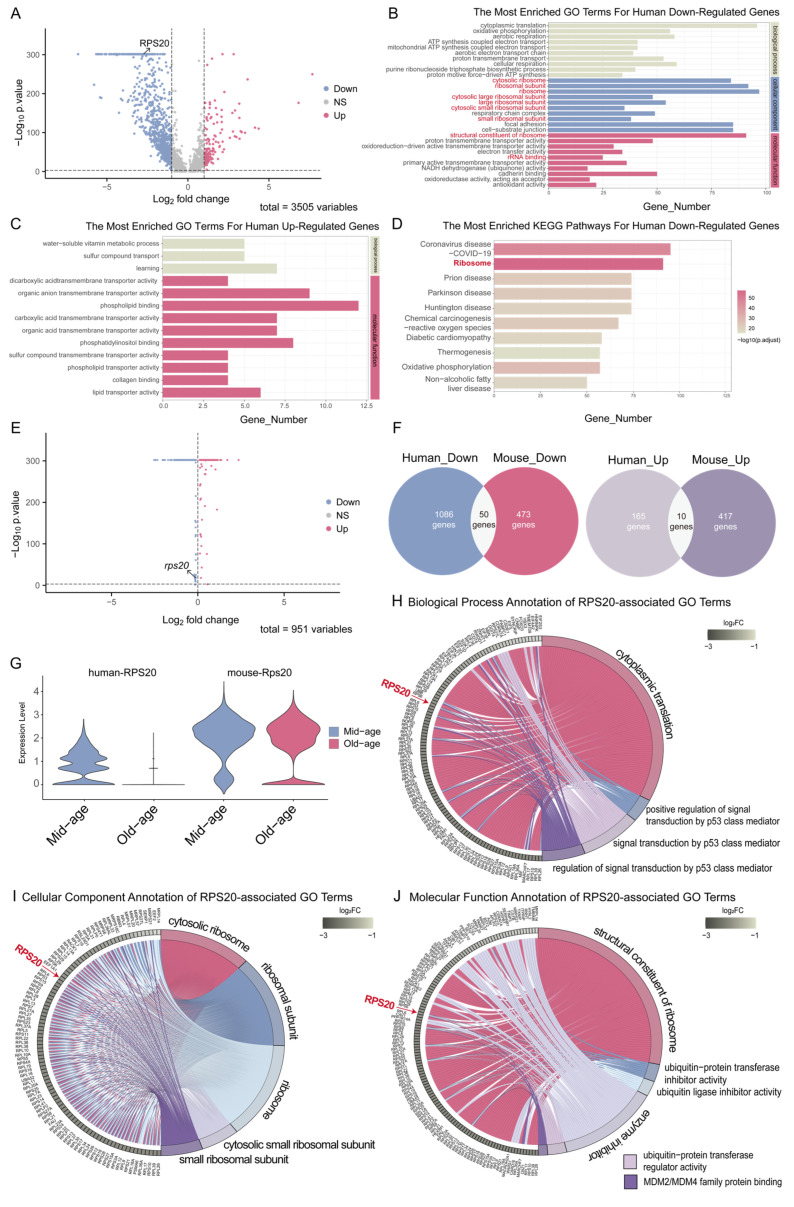
Cross-species bioinformatics identified conserved aging signatures in cerebrovascular endothelial cells. (**A**) Volcano plots showing the differentially expressed genes in mid- and old-aged human cerebrovascular endothelial cells; (**B**) GO analysis of down-regulated genes in aged human cerebrovascular endothelial cells; (**C**) GO analysis of up-regulated genes in aged human cerebrovascular endothelial cells; (**D**) KEGG analysis of down-regulated genes in aged human cerebrovascular endothelial cells; (**E**) Volcano plots showing the differentially expressed genes in mid- and old-aged mouse cerebrovascular endothelial cells; (**F**) Wayne diagrams showing the overlapped up and down-regulated genes in aged human and mouse cerebrovascular endothelial cells; (**G**) Expression of *rps20* in human and mouse cerebrovascular endothelial cells in mid-aged and old-aged adults; (**H**–**J**) Genes and signaling pathways involved in the *rps20* participated GO terms.

Differentially expressed genes (DEGs) between middle- and old-aged human brain endothelial cells were identified using the criteria described in the Methods (Figure 1A). The DEGs contained 165 up-regulated genes and 1086 down-regulated genes associated with aging. Gene Ontology (GO) enrichment analysis revealed that aging-related down-regulated DEGs were significantly enriched in ribosome-related biological processes (Figure 1B), whereas up-regulated DEGs were associated with pathways involved in water-soluble vitamin metabolism, transmembrane transport, and phospholipid/collagen binding activity (Figure 1C). KEGG pathway analysis further confirmed that the ribosomal pathway was enriched in down-regulated genes (Figure 1D). Together, these results support that ribosomal pathways play critical roles in aged brain endothelial cells.

To investigate the evolutionary conservation of RP gene expression during aging, we further identified DEGs in mouse brain endothelial cells (Figure 1E). Cross-species comparison yielded 50 conserved down-regulated and 10 up-regulated genes during aging (Figure 1F and Figure S1A). Interestingly, only two RP genes—RPS20 (Figure 1G) and RPL10 (Figure S1B)—showed consistent downregulation across both species. We focused on investigating the role of *rps20* in brain vascular development.

### 3.2. rps20 Protein Is Highly Evolutionarily Conserved in Vertebrates

**Figure 2:**
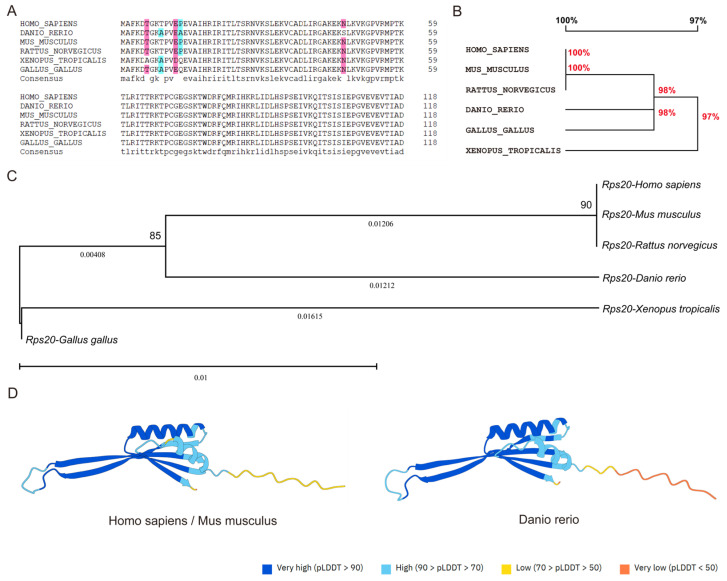
Evolutionary conservation of RPS20 protein in vertebrates. (**A**) Multiple sequence alignment analysis of RPS20 protein sequences from six representative species. Red sequences indicate homology level ≥75% and blue sequences indicate homology level ≥50%; (**B**) Homologous tree constructed based on the multiple sequence alignment of RPS20; (**C**) Phylogenetic tree of the RPS20 protein showing the high evolutionary conservation of RPS20 protein; (**D**) 3D structures of human, mouse, and zebrafish RPS20 proteins predicted by AlphaFold. pLDDT (per-residue confidence score) indicating residue-level confidence of AlphaFold-predicted structures.

To assess the evolutionary conservation of the RPS20 protein across vertebrates, we performed a multiple sequence alignment using RPS20 protein from *Homo sapiens*, *Mus musculus*, *Rattus norvegicus*, *Danio rerio*, *Xenopus tropicalis*, and *Gallus gallus* (Figure 2A). The results demonstrated that the RPS20 sequences of *Homo sapiens*, *Mus musculus* and *Rattus norvegicus* are identical. Additionally, RPS20 from *Danio rerio* and *Gallus gallus* shares 98% homology with human protein, while *Xenopus tropicalis* shows 97% homology (Figure 2B). Meanwhile, a phylogenetic tree constructed using the MEGA further confirmed the high evolutionary conservation of RPS20 protein among these species (Figure 2C). Given that protein function is closely related to the three-dimensional (3D) structures, we next predicted the 3D structures of human and zebrafish RPS20 proteins using AlphaFold [21]. The results demonstrated that both RPS20 proteins are composed of α-helixes, β-folds, and random coils according to PSSpred, and they exhibit a high degree of structural similarity (Figure 2D) [22].

### 3.3. rps20 Is Highly Enriched in the Zebrafish Brain During Early Development

**Figure 3:**
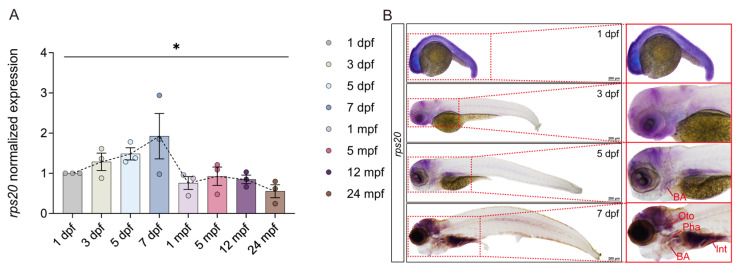
Spatiotemporal expression analysis of *rps20* in zebrafish. (**A**) Relative expression of *rps20* in the brain or whole embryo at eight time points in the zebrafish life cycle. Relative expression assays were normalized using 1 dpf as a baseline for three replicate experiments. *n* = 3. Data presented as mean ± SEM. Statistical significance determined by one-way ANOVA (* *p* < 0.05); (**B**) Spatiotemporal expression of *rps20* at four time points of zebrafish embryonic development. BA: branchial arch; Oto: Otolith; Pha: Pharynx; Int: Intestine; mpf, months post fertilization. Scale bar, 200 μm.

To comprehensively investigate the expression dynamics of *rps20* in the zebrafish brain, we conducted qPCR analysis on brain tissues across eight time points, spanning early embryogenesis to adulthood and aging. The results showed a bell-shaped expression pattern: *rps20* expression progressively increased during early development (1–7 dpf), peaking at 7 dpf, followed by a gradual decline from 1 mpf to 24 mpf (Figure 3A). The temporal expression pattern suggests *rps20* is actively involved in early developmental processes. To further characterize the spatiotemporal expression of *rps20*, we performed whole-mount in situ hybridization (WISH) at 1, 3, 5, and 7 dpf. At 1 dpf, *rps20* was ubiquitously expressed throughout the embryo. By 3 dpf, the signals became more restricted to the head. At 5 dpf, specific enrichment emerged in the brain and branchial arches. By 7 dpf, *rps20* expression was localized to the brain, otoliths, pharynx, branchial arches, and intestine, with particularly intense signals in the developing brain (Figure 3B).

### 3.4. Knockout of rps20 Retards Hindbrain Vascular Development

To investigate the potential function of *rps20* in zebrafish cerebrovascular development, we knocked out *rps20* with CRISPR/Cas9 and examined the cerebrovascular development. We designed a single-guide RNA (sgRNA) targeting exon 2 of *rps20*. The sequencing results confirmed efficient *rps20* mutagenesis by this sgRNA (Figure S2A–C). Phenotypically, *rps20*-KO zebrafish exhibited significantly shortened body lengths at both 3 dpf and 5 dpf (Figure S3B,C). Given the association of *rps20* with aging, we assessed the expression of canonical aging -related markers—*pcna* [23,24], *p15* [25,26], and *p53* [27]—via qPCR. The results showed *rps20* deficiency significantly up-regulated *p15* and *p53*, while *pcna* expression was markedly reduced, indicating impaired proliferative capacity and cell cycle progression (Figure S2D–F).

**Figure 4:**
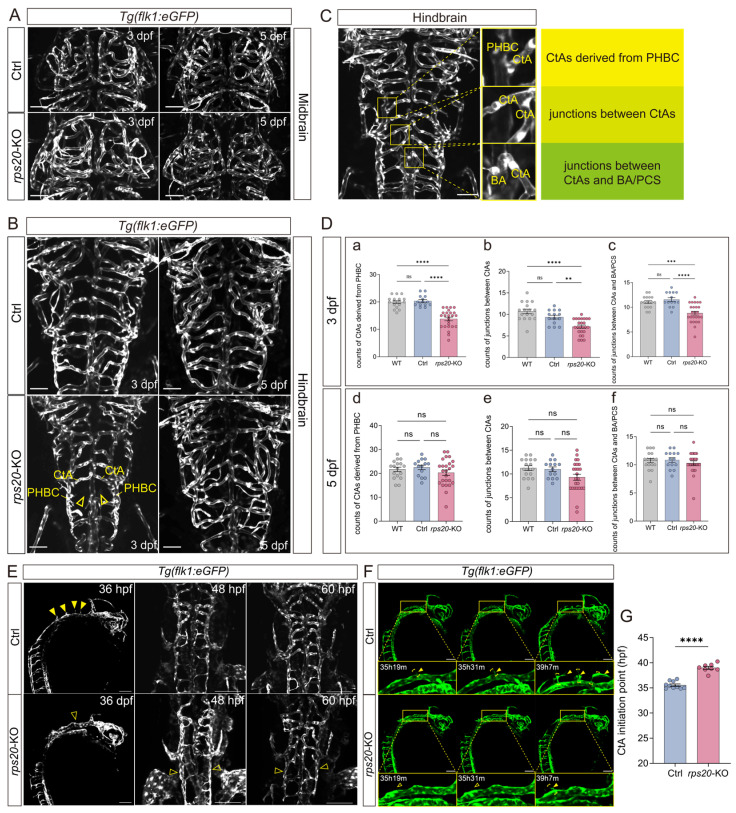
Knockout of *rps20* impaired hindbrain vascular development. (**A**) Representative images showing the midbrain vasculature at 3 dpf and 5 dpf; (**B**) Representative images showing the hindbrain vasculature at 3 dpf and 5 dpf; (**C**) Schematic representation of three quantitative parameters for quantifying hindbrain vascular development; (**D**) Quantitative analysis of hindbrain vasculature at 3 dpf and 5 dpf. At 3 dpf, counts of CtAs derived from PHBC: WT: 19.84 ± 2.1, Ctrl: 20.36 ± 1.9, *rps20*-KO:13.8 ± 3.4; counts of junctions between CtAs: WT: 10.71 ± 2.1, Ctrl: 9.36 ± 1.7, *rps20*-KO: 7.16 ± 1.8; counts of junctions between CtAs and BA/PCS: WT: 11.06 ± 1.3, Ctrl: 11.57 ± 1.6, *rps20*-KO: 8.8 ± 2.1. In both 3 dpf and 5 dpf, 17 WT larvae, 14 Ctrl larvae, and 25 *rps20*-KO larvae were analyzed; (**E**) Representative images showing the developmental tracking of central arteries (CtAs). Solid yellow arrowheads indicate sprouting CtAs. Empty yellow arrowheads indicate absence of CtAs; (**F**) Time-lapse imaging of CtAs sprouting dynamics. Solid yellow arrowheads indicate sprouting CtAs. Empty yellow arrowheads indicate absence of CtAs; (**G**) Initiation timing of CtAs in control and *rps20* knockout zebrafish, 10 sprouting events in ctrl larvae, and 8 sprouting events in *rps20*-KO larvae were analyzed. Scale bars, 50 μm (**A**,**B**), 100 μm (**E**,**F**). Data presented as mean ± SEM. Statistical significance determined by one-way ANOVA with Tukey’s Honestly Significant Difference test in figure (**D**). Statistical significance determined by unpaired Student’s *t*-test in (**G**) (** *p* < 0.01, *** *p* < 0.001, **** *p* < 0.0001, ns = no significance).

Next, we examined the effect of *rps20* deficiency on brain vascular development using the *Tg(flk1:eGFP)* zebrafish line, in which endothelial cells were labeled with eGFP. To rule out Cas9 toxicity, Cas9 protein-only injected zebrafish were used as controls. At 3 dpf and 5 dpf, no significant vascular abnormalities were observed in the midbrain of *rps20*-KO larvae (Figure 4A and Figure S3A). In contrast, the hindbrain vasculature was markedly impaired, with impaired angiogenic sprouting of central arteries (CtAs) from the primordial hindbrain channel (PHBC) at 3 dpf (Figure 4B and Figure S3A).

To quantitatively assess hindbrain CtAs development, we measured three parameters: (1) Counts of CtAs derived from PHBC; (2) Counts of junctions between CtAs; (3) Counts of junctions between CtAs and BA/PCS [28] (Figure 4C). The results showed Cas9 protein alone did not adversely affect CtA development, compared to the WT zebrafish larvae. However, in *rps20*-KO zebrafish larvae, all three parameters were significantly reduced compared to Ctrl at 3 dpf, though these defects largely recovered by 5 dpf (Figure 4D).

To explore the cellular dynamics underlying the delayed CtA development, we performed in vivo time-lapse imaging of CtAs at 1.5 dpf, 2 dpf, and 2.5 dpf, based on the developmental timeline of zebrafish hindbrain vasculature [28,29]. In ctrl larvae, CtAs began sprouting from the PHBC by 1.5 dpf. In contrast, *rps20*-KO larvae exhibited delayed sprouting, with a noticeable developmental lag through 2.5 dpf (Figure 4E). Furthermore, tracking individual CtA sprouting events with time-lapse live imaging revealed that CtA sprouting onset in ctrl larvae occurred at ~35 h post-fertilization (hpf), whereas *rps20*-KO siginificantly delayed the sprouting initiation until ~39 hpf (Figure 4F,G).

We further investigated whether *rps20* affects vascular development outside the brain by examining the development of ISVs. We found that, although the ISVs showed as normal between ctrl and *rps20*-KO zebrafish larvae after 2 dpf, *rps20* knockout also significantly decreased ISV sprouting at 1 dpf (Figure S3D,E). These results are consistent with the high and ubiquitous expression of *rps20* at 1dpf (Figure 3B), supporting its early developmental role.

To determine whether the overexpression of *rps20* affected cerebrovascular development, we generated a vascular endothelial-specific *rps20*-overexpressing zebrafish, *Tg(flk1: rps20-P2A-mApple)* (Figure S4A). At both 3 dpf and 5 dpf, no significant changes in hindbrain or trunk vasculature were observed in *rps20*-overexpressed zebrafish larvae compared to controls (Figure S4B–D). Likewise, body length remained unchanged (Figure S4E,F).

### 3.5. Knockout of rps20 Causes Intracerebral Hemorrhage

**Figure 5:**
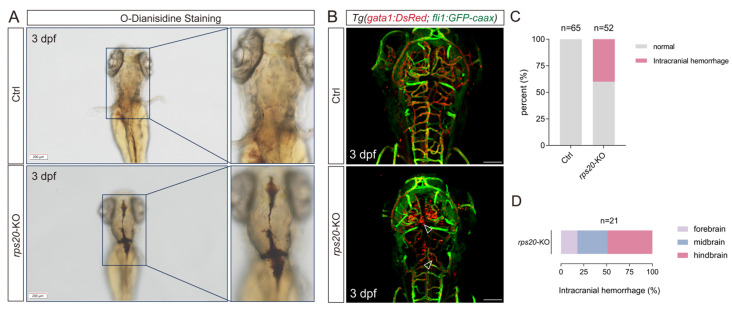
Knockout of *rps20* caused intracranial hemorrhage. (**A**) Representative images of *O*-dianisidine staining of *rps20* knockout and control zebrafish; (**B**) Representative images of intracranial hemorrhage of *rps20* knockout zebrafish with dual-labeled vasculature and erythrocytes. Empty arrowheads indicate sites of erythrocyte aggregation outside the cerebral blood vessels; (**C**) Intracranial hemorrhage incidence statistics in control versus *rps20*-KO zebrafish. 65 WT larvae and 52 *rps20*-KO larvae were analyzed; (**D**) Regional hemorrhage distribution analysis (forebrain/midbrain/hindbrain) in *rps20*-KO zebrafish. A total of 21 *rps20*-KO larvae with intracranial hemorrhage were analyzed. Scale bar, 200 μm (**A**). Scale bar, 100 μm (**B**).

The development of brain vasculature is closely followed by the maturation of the blood–brain barrier, which is essential for maintaining the brain microenvironment’s homeostasis. Notably, we found ~40% of *rps20*-KO zebrafish larvae exhibited intracranial hemorrhage compared to the ctrl zebrafish larvae (Figure 5A,C). To further validate the cerebral hemorrhage phenotype, we performed *O*-dianisidine staining [20]. The staining revealed significant accumulation of erythrocytes within the brain parenchyma of *rps20*-KO larvae (Figure 5A), indicating hemorrhagic leakage beyond the vasculature. Furthermore, we employed the *Tg (gata1: DsRed; fli1: GFP-caax)* transgenic zebrafish line, in which the erythrocytes and endothelial cells were labeled with DsRed and GFP-caax, to more precisely visualize hemorrhagic event. We found widespread cerebral hemorrhage accompanied by a dramatic reduction in intravascular erythrocyte density in *rps20*-KO larvae, further supporting the compromised vascular integrity (Figure 5B). The quantification of the regional distribution of hemorrhage showed that 49% of hemorrhagic events occurred in the hindbrain, 33% in the midbrain, and 18% in the forebrain (Figure 5D). This regional distribution correlated with the defect of hindbrain vascular development in *rps20*-KO larvae.

### 3.6. Knockout of rps20 Decreased the Angiogenesis-Related Genes

**Figure 6:**
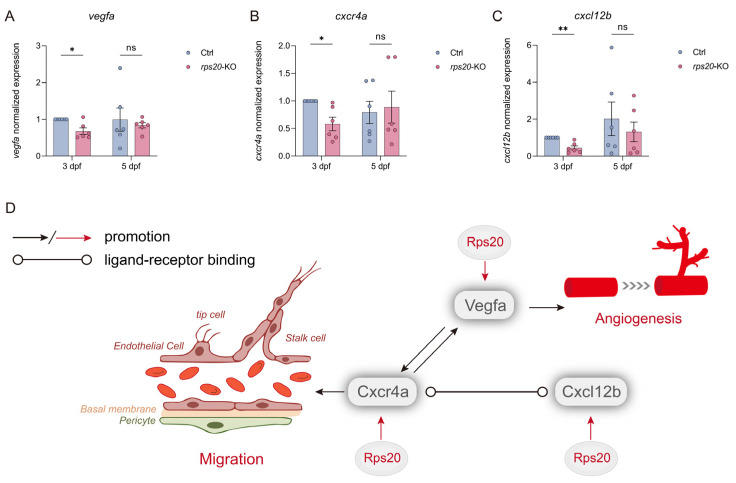
Knockout of *rps20* decreased vascular development-related genes. (**A**–**C**) RT-qPCR analysis of *vegfa* (**A**), *cxcr4a* (**B**), and *cxcl12b* (**C**) mRNA levels in *rps20* knockout and ctrl embryos at 3 dpf and 5 dpf; *n* = 6. (**D**) Schematic diagram of *rps20* regulating vascular development through *vegfa*, *cxcr4a*, and *cxcl12b*. Arrows denote promoting effects or activation. Circle-ended lines represent ligand–receptor binding interactions. Data presented as mean ± SEM. Statistical significance determined by multiple paired *t*-tests (* *p* < 0.05, ** *p* < 0.01, ns = no significance).

To elucidate the molecular mechanisms underlying *rps20* deficiency-induced hindbrain cerebrovascular defects, we examined the expression of key regulators of angiogenesis, including *vegfa*, *cxcr4a*, and *cxcl12b*. Quantitative RT-qPCR revealed a significant downregulation of *vegfa*, *cxcl12b*, and *cxcr4a* in *rps20*-KO larvae at 3 dpf compared to controls. However, by 5 dpf, expression levels of these genes were no longer significantly different (Figure 6A–C), coinciding with the recovery of hindbrain vascular development. Thus, we assume that Rps20 modulates the expression of Vegfa, Cxcr4a, and Cxcl12b, either through direct or indirect mechanisms, thereby contributing to early cerebrovascular development (Figure 6D).

## 4. Discussion

Rps20 protein is essential for ribosome assembly, ensuring the proper folding of the ribosomal small subunit [30,31,32,33]. Beyond its canonical ribosomal functions, RPS20 has been implicated in the pathogenesis of multiple diseases. For example, RPS20 drives clear cell renal cell carcinoma (ccRCC) progression by co-activating the AKT-mTOR and ERK-MAPK signaling cascades [14]. Furthermore, germline mutations in RPS20 cause predisposition to hereditary nonpolyposis colorectal carcinoma [34]. In the central nervous system, RPS20 serves as a prognostic marker in glioblastoma [35] and medulloblastoma [36] while also acting as a biomarker and potential therapeutic target in Alzheimer’s disease [37,38] and autoimmune hepatitis [39]. Additionally, RPS20 facilitates viral protein biosynthesis by suppressing host immune activity during infections [40,41,42]. Other ribosomal proteins, such as *rps3* [43], *rps5* [44] and *rps27a*, also perform diverse extra ribosomal functions, underscoring the broader biological significance of ribosomal proteins beyond translation.

In this study, through the in-depth analysis of scRNA-seq data from brain vascular endothelial cells from mid- and old-aged humans and mice, we identified many genes attaching to the ribosome-associated GO and KEGG terms, and a substantial proportion of these terms comprised genes encoding ribosomal protein (RP). Previous studies have shown that specific RPs—such as RPL5, RPL7, RPL11, RPS20, and RPS23—regulate the cell cycle by the stabilization of P53 through inhibiting MDM2 [13]. Additionally, Frédéric et al. demonstrated that RPS14 accumulation in senescent cells inhibits CDK4 activity, blocking Rb phosphorylation and inducing cell cycle arrest and cell senescence [45]. Together, these results support that ribosomal pathways play critical roles in aged brain endothelial cells.

To ensure that the research possesses human-relevant translatability and to preliminarily justify the feasibility of conducting *rps20* functional experiments in zebrafish, we analyzed the evolutionary conservation of *rps20* in vertebrates. The results indicate that it is highly conserved among vertebrates, which lends support to our subsequent research plans.

Through qPCR and whole-mount in situ hybridization (WISH) analysis of the spatiotemporal expression of *rps20* at various stages of zebrafish development, it was revealed that *rps20* exhibited strong expression activity during the early embryonic development of zebrafish. Notably, it was highly enriched in the head region, suggesting a potential role in the early development of head-related tissues. However, due to the resolution limitations of the staining observation, we were unable to observe the specific expression pattern of *rps20* in the cerebral blood vessels using WISH.

In the functional study, knocking out *rps20* shortened zebrafish body length, likely due to inhibited embryonic cell proliferation. Similarly, reduced body size due to *rps20* deficiency has also been observed in studies of mice [46] and Angus cattle [47]. As *rps20* is vascular aging-related, which involves permanent cell cycle arrest with changes in markers like *pcna* [23,24], *p15* [25,26], and *p53* [27], we measured their expression. Results showed decreased *pcna* and increased *p15* and *p53* expression, which confirmed cell cycle inhibition. This suggests that *rps20* knockout may cause zebrafish embryonic premature aging, warranting further study. Intriguingly, a previous report showed that Rps20 stabilizes P53 proteins by inhibiting MDM2-mediated ubiquitination, thereby promoting cell cycle arrest [13], which is paradoxical to our findings at the mRNA level. This discrepancy may reflect the existence of feedback regulatory or context-dependent, multi-level regulation of *p53* expression and activity by Rps20 under different physiological conditions.

Then, we focused on the brain vascular development of the zebrafish midbrain and hindbrain. Spatially, the zebrafish midbrain is demarcated as the region between the anterior cerebral vein (ACeV) and middle cerebral vein (MCeV), while the hindbrain spans from the MCeV to the anterior boundary of the dorsal longitudinal anastomotic vessel (DLAV) (Figure S3A). We found that *rps20*-knockout zebrafish embryos exhibited an absence of hindbrain vasculature (CtA) at 3 dpf, while midbrain vascular development showed no significant impact. During zebrafish hindbrain vascular development, the PHBC forms first via vasculogenesis, followed by the migration of endothelial cells from the PHBC toward the midline to form the basilar artery (BA). Subsequent, the CtAs, sprouting from the PHBC, connect with either the BA or posterior communicating segment (PCS) [29,48]. Based on the developmental process of the hindbrain vasculature in zebrafish, we quantified the development of hindbrain vasculature in *rps20* deficient zebrafish and found that *rps20* was required for normal hindbrain vascular development. Based on our continuous tracking of CtA sprouting, we ultimately discovered that *rps20* deficiency caused delayed CtA sprouting, indicating that *rps20* participates in the early sprouting process of the zebrafish CtA. Concurrently, intersegmental vessels (ISVs) on the trunk of *rps20*-knockout embryos also exhibited absence at 1 dpf, which was consistent with the strong *rps20* expression in the zebrafish trunk at 1 dpf (Figure 3B). Interestingly, although *rps20* deficiency impaired hindbrain vascular development at early stages, these defects were restored at later developmental stages. Given the functional redundancy among ribosomal proteins, including *rps20*, *rps10*, *rps14*, *rps15,* etc., it is plausible that other ribosomal proteins may compensate for the loss of *rps20*, facilitating developmental recovery. Importantly, the endothelial-specific overexpression of *rps20* didn’t enhance the brain vascular development, suggesting a threshold or saturation effect in physiological Rps20 levels [12,49,50]. Collectively, these results demonstrate *rps20*-mediated regulation of early cerebrovascular development roles is tightly controlled.

The development of brain vasculature is closely followed by the maturation of the blood–brain barrier, which is essential for maintaining the brain microenvironment’s homeostasis. *rps20* knockout induced cerebral hemorrhage in zebrafish embryos, with the hindbrain being the primary site of bleeding. This regional distribution correlated with the defect of hindbrain vascular development in *rps20*-knockout larvae. These findings suggest that *rps20* is critical for establishing and maintaining cerebrovascular integrity, and its loss compromises vascular barrier function, particularly in regions undergoing delayed vascular development.

Finally, regarding the mechanisms underlying *rps20*’s involvement in zebrafish hindbrain vascular development, *Vegfa* is a master regulator of vascular development, critically governing angiogenesis, vasculogenesis, and vascular branching [51,52]. The CXCR4a–CXCL12b signaling axis orchestrates endothelial cell migration, spatial patterning, and vascular network formation through chemotactic gradients during embryogenesis [53,54]. Notably, these pathways often cooperatively regulate endothelial cell proliferation and sprouting in zebrafish hindbrain vascular development [55,56,57]. Previous studies have shown that VEGFA can upregulate the expression of CXCR4a in vascular endothelial cells [58]. Concurrently, the CXCR4a–CXCL12b signaling axis can also enhance VEGFA expression through the PI3K/Akt signaling pathway [59], indicating a positive feedback loop between these pathways. Building upon these findings and our current results, we proposed that Rps20 modulates the expression of Vegfa, Cxcr4a, and Cxcl12b, either through direct or indirect mechanisms, thereby contributing to early cerebrovascular development (Figure 6D).

## 5. Conclusions

Overall, our study demonstrates that *rps20* participates in cerebrovascular development and vascular homeostasis maintenance in zebrafish. Mechanistically, we found that the critical signaling pathways essential for hindbrain vascular development—including VEGF, CXCL12b, and CXCR4A—were significantly decreased in *rps20*-KO zebrafish. This finding proves that aging-related genes can also be involved in vascular developmental processes, which aligns with the findings about the well-known aging-associated gene p16Ink4a in endothelial cells during development and aging [60,61], collectively offering a novel perspective for vascular development research.

However, there are still some limitations and unresolved issues. First, regarding human relevance, it is worthwhile to examine the functions of *rps20* in the brain vascular development in humans or mice. Second, compensatory pathways in vascular development following *rps20* knockout in zebrafish warrant deeper exploration. The function of the identified aging-associated ribosomal gene *rpl10* in angiogenesis also merits dedicated research. Third, further functional studies of *rps20* in angiogenesis at the cellular level would enhance understanding of its role in vascular development.

## Figures and Tables

**Table 1:** R packages and versions.

R-Packages	Version
Seurat	5.0.1
clusterProfiler	4.10.0
EnhancedVolcano	1.20.0
ggplot2	3.4.4

**Table 2:** qPCR primers.

Gene	Forward	Reverse
*β-actin*	CGAGCAGGAGATGGGAACC	CAACGGAAACGCTCATTGC
*rps20*	CTGACCAGCCGTAACGTCAA	GATGCGGAGGGTCTTGGTAG
*pcna*	AGGAGGATGAAGCGGTAACAA	GTCTTGGACAGAGGAGTGGC
*p15*	GAGGATGAACTGACCACAGCA	CAAGAGCCAAAGGTGCGTTAC
*p53*	AGGTCTTTTGAGGTGCGTGT	AGAAGATTCTTTCACCAAACTACG
*cxcl12b*	CCCAGAGACTGACGCAAAGC	TTGGGTTGATGCAGACCTCTC
*cxcr4a*	TGGCTTATTACGGACACATCGT	CCGTACACCGTTGGGAGAAA
*vegfa*	TCAAAGCAAAGAAAGAAAACCACTG	ATTTGCAGGAGCATTTACAGGTG

**Table 3:** *O*-Dianisidine staining solution.

Component	Dosage
*O*-Dianisidine (Sigma-Aldrich, Darmstadt, Germany)	3 mg
3M NaAc, PH 5.2 (Beyotime, Shanghai, China)	16.5 μL
H_2_O_2_	108.5 μL
Ethanol	2 mL
H_2_O	To 5 mL

## Data Availability

The data are contained within this article.

## Supplementary Materials

The following supporting information can be downloaded at: https://www.mdpi.com/article/10.3390/cells14141070/s1. Figure S1. Expression heatmap of genes in cerebrovascular endothelial cells of mid-aged and old-aged humans and RPL10 expression. Figure S2. Design and validation of *rps20* knockout and expression of aging-associated genes. Figure S3. Morphological and trunk vascular effects in *rps20*-KO zebrafish larvae. Figure S4. Endothelial-specific *rps20* overexpression maintains normal vascular and body development.

## Abbreviations

The following abbreviations are used in this manuscript:

ACeV	anterior cerebral vein
BA	basilar artery
BBB	blood–brain barrier
ccRCC	clear cell renal cell carcinoma
CDS	coding sequence
CtA	central artery
DEG	Differentially Expressed Gene
DLAV	dorsal longitudinal anastomotic vessel
dpf	day post fertilization
GFP	Green Fluorescent Protein
GO	Gene Ontology
hpf	hour post-fertilization
KEGG	Kyoto Encyclopedia of Genes and Genomes
MCeV	middle cerebral vein
mpf	month post fertilization
PCS	posterior communicating segment
PFA	paraformaldehyde
PHBC	primordial hindbrain channel
RP	Ribosomal protein
WISH	whole-mount in situ hybridization
