# Supplementary material for: Regulation of Hindbrain Vascular Development by rps20 in Zebrafish
Source: Cells. 2025 Jul 13;14(14):1070. doi: 10.3390/cells14141070 (PMC12293849; doi:10.3390/cells14141070)
Supplement: Supplementary file 1 [file cells-14-01070-s001.zip › cells-3719061-supplementary.pdf]

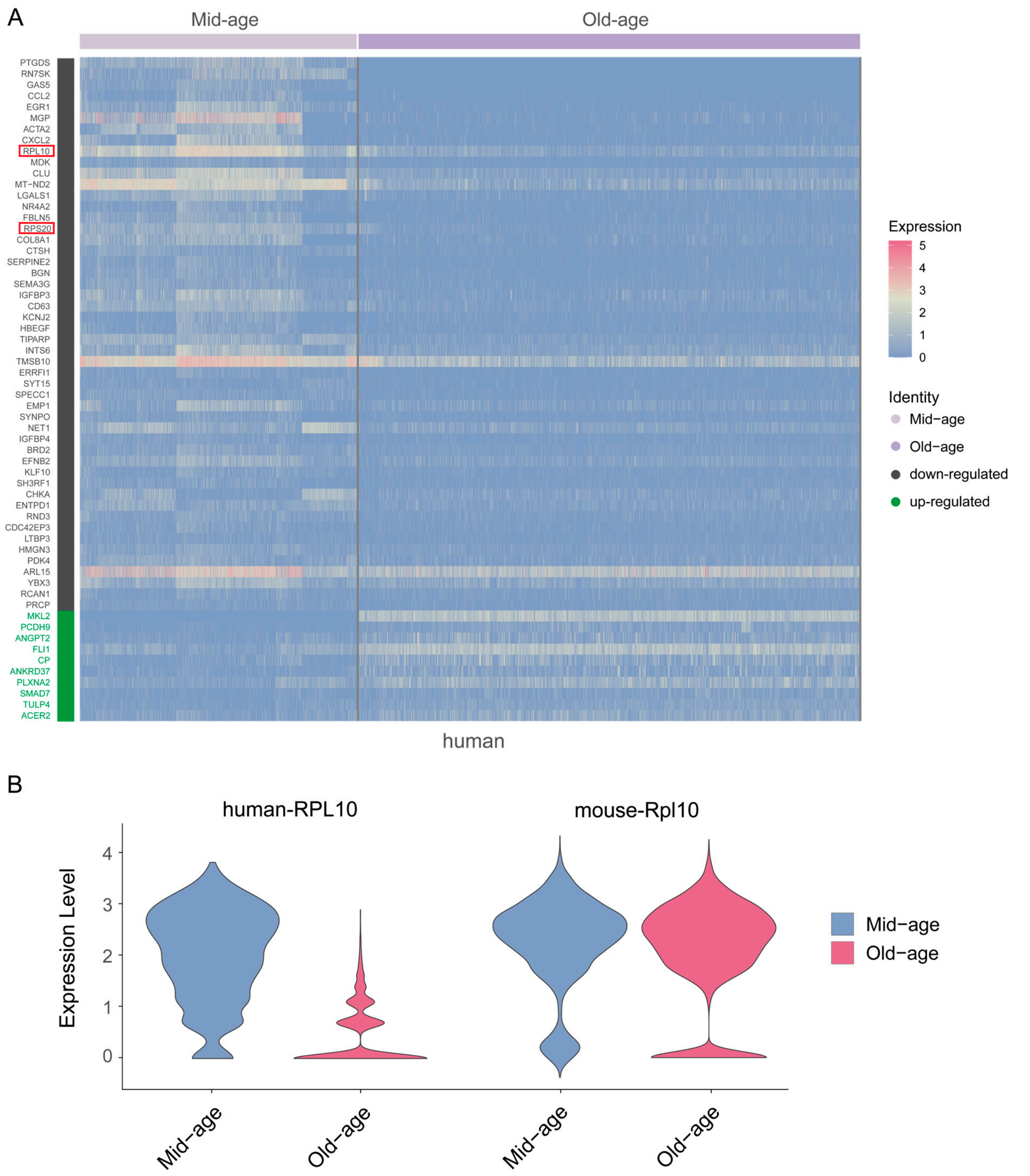

Figure S1. Expression heatmap of genes in brain vascular endothelial cells of middle-aged and old-aged human and RPL10 expression. (**A**) Expression heatmap of genes in cerebrovascular endothelial cells. RPL10 and RPS20 are highlighted with red boxes. The dark gray section represents 50 shared downregulated genes in the aging process of human and mouse brain vascular endothelial cells, while the green section indicates 10 shared upregulated genes; (**B**) Expression of RPL10 in human and mouse brain vascular endothelial cells in mid-aged and old-aged adults.

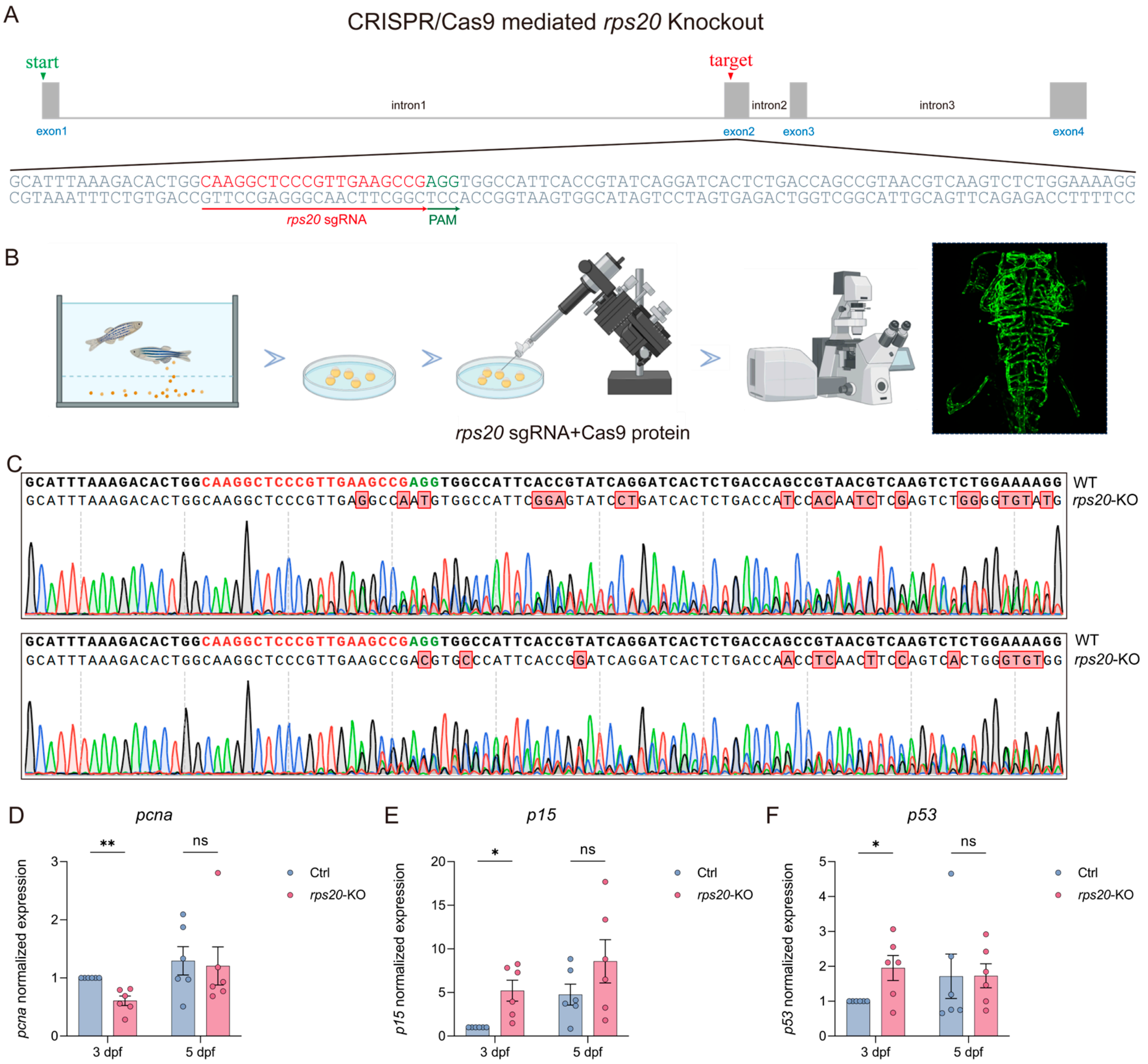

Figure S2. Design and validation of *rps20* knockout and expression of aging-associated genes. (**A**) Gene structure of zebrafish *rps20* and sgRNA target design. Red text indicates sgRNA-targeting DNA sequence; green text denotes downstream PAM sequence; (**B**) Experimental workflow for *rps20* knockout in zebrafish; (**C**) Sequencing comparison of *rps20* between knockout and WT zebrafish; (**D-F**) Relative expression levels of *pcna*, *p15* and *p53* in *rps20* knockout and control zebrafish at 3 dpf and 5 dpf. Expression levels of both control and knockout groups at 3 dpf and 5 dpf were normalized to the corresponding control group at 3 dpf for each gene.

Data presented as mean ± SEM. Statistical significance determined by multiple paired t-tests (*p<0.05, **p<0.01).

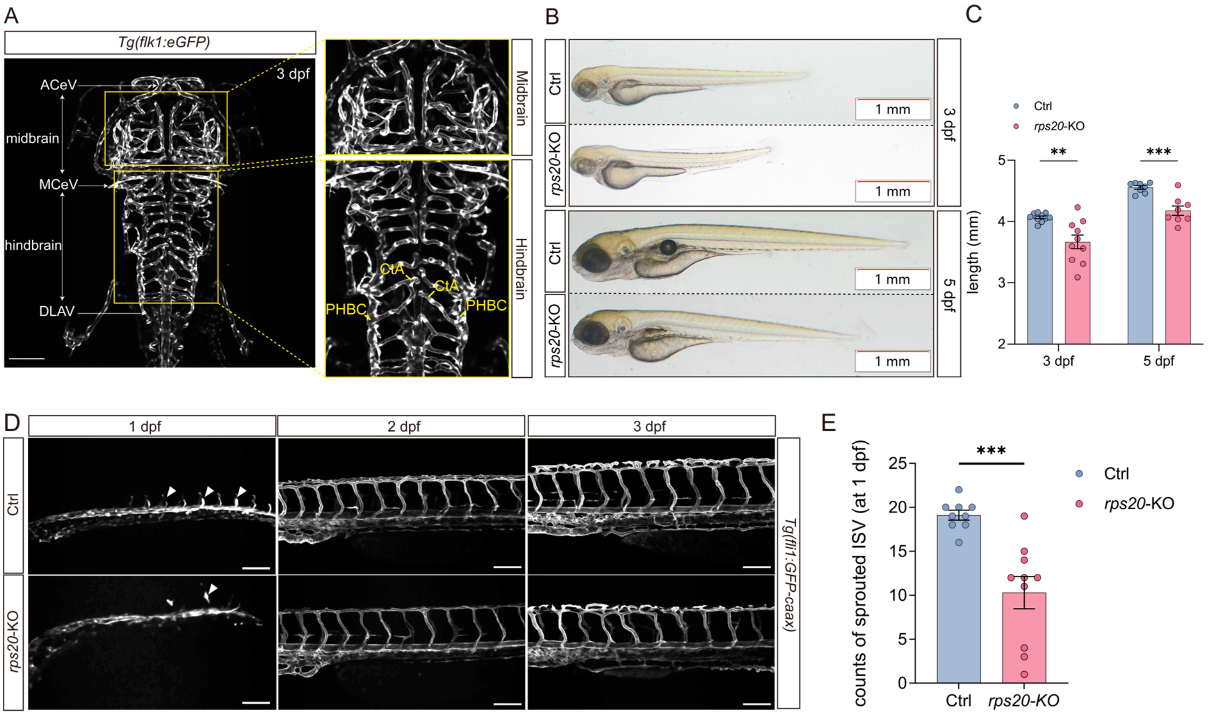

Figure S3. Morphological and trunk vascular effects in *rps20*-KO zebrafish larvae. (**A**) Anatomical demarcation of midbrain and hindbrain regions in zebrafish; (**B**) Bright-field images of *rps20*-KO and control larvae at 3 dpf and 5 dpf; (**C**) Body length quantification of *rps20*-KO versus control zebrafish larvae at 3 dpf and 5 dpf. At 3 dpf, 10 control larvae, 10 *rps20*-KO larvae were analyzed; at 5 dpf, 8 control larvae, 8 *rps20*-KO larvae were analyzed; (**D**) Images of ISVs in *rps20*-KO zebrafish larvae at 1, 2, and 3 dpf. ISV sprouts were indicated by arrowheads; (**E**) Quantification of ISV sprouts at 1 dpf in *rps20*-KO and control zebrafish larvae. 9 control larvae, 10 *rps20*-KO larvae were analyzed. Scale bar, 100 μm (A, E). Scale bar, 1 mm (B). Data presented as mean ± SEM. Statistical significance determined by multiple paired t-tests in figure C. Statistical significance determined by unpaired Student's t-test in figure E (**p<0.01, ***p<0.001).

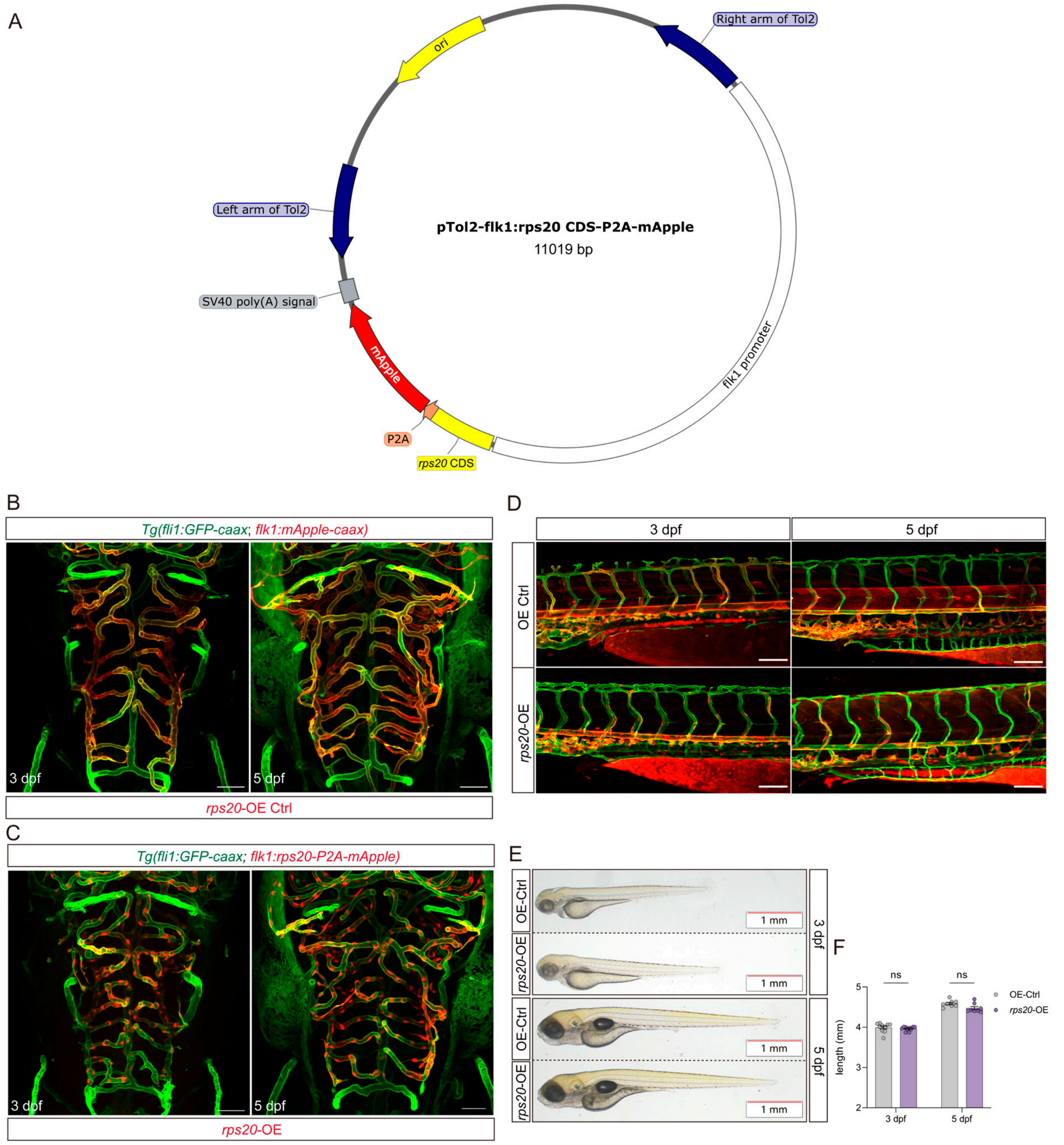

Figure S4. Endothelial-specific *rps20* overexpression maintains normal vascular and body development. (**A**) Plasmid map for constructing vascular endothelial-specific *rps20* overexpression. (**B**) Hindbrain vascular images in control zebrafish at 3 and 5 dpf. (**C**) Hindbrain vascular images in *rps20*-OE zebrafish larvae at 3 and 5 dpf. (**D**) Images of ISVs in *rps20*-OE and control zebrafish larvae at 3 and 5 dpf. (**E**) Bright-field images of *rps20*-OE and control zebrafish larvae at 3 and 5 dpf. (**F**) Body length quantification of *rps20*-overexpressing versus control zebrafish at 3 and 5 dpf. At 3 dpf, 10 control larvae, 10 *rps20*-KO larvae were analyzed; at 5 dpf, 8 control larvae, 8 *rps20*-KO larvae were analyzed. Scale bar, 50 μm (B, C); 100 μm (D); 1 mm (E). Data presented as mean ± SEM. Statistical significance determined by unpaired Student's t-test (ns, non-significant, p>0.05).
